# A platform for investigating prompt framing as interface parameters in foundation models for robotics

**DOI:** 10.3389/frobt.2026.1771992

**Published:** 2026-04-22

**Authors:** Anup Tuladhar, Eli Kinney-Lang

**Affiliations:** 1 SunyataTek Inc, Calgary, AB, Canada; 2 Department of Biomedical Engineering, University of Calgary, Calgary, AB, Canada; 3 Azrieli Accelerator, University of Calgary, Calgary, AB, Canada

**Keywords:** cognitive robotics, robotic control, hybrid control algorithm, foundation models, large language models (LLM), prompt engineering, reinforcement learning, sequential decision making

## Abstract

Foundation models, in particular large language models (LLMs), are finding increasing popularity when used in describing goals for robotic control, decision making, and execution. Recently, proposals for hybrid paradigms leveraging strengths of reinforcement learning (RL) agents in tandem with LLMs for robotic control have been demonstrated. The interface between the RL agents and the language model however offers a unique opportunity to explore how prompt framing may affect such hybrid systems. This work presents a controlled experimental platform to measure and better understand how manipulation of the interface between RL agents and an LLM impacts behaviour of a hybrid advisor-arbiter architecture. We compared three agents under matched evaluation protocols and initializations in a simulated navigation environment: (i) RL-only tabular Q-learning; (ii) LLM-only (stateless) action selection; and (iii) a hybrid LLM + RL agent. Under a constrained interaction budget (10 episodes per world), the hybrid LLM + RL agent achieves higher mean success and higher mean cumulative reward than both RL-only and LLM-only baselines. Advisor-channel ablations (random recommendations and null recommendations) reduce performance, indicating that structured advice contributes beyond adding extra text. We further demonstrate prompt framing as a controlled factor by evaluating navigation-role personas, narrative personas, and relational variants of a caregiver prompt under matched conditions, yielding heterogeneous effects across framings. The contribution of this work is to provide a structured testbed and evaluation approach for investigating the impact of prompt framing on multi-step decision making and control tasks.

## 1 Introduction

Foundation models have become a core pillar of research in many fields exploring artificial intelligence: from neuroscience (Moor et al., 2023; Zhou et al., 2025; Tak et al., 2026) and brain-computer interfaces (Yue et al., 2024) to natural language processing and wireless communications (Alikhani et al., 2024). In the field of robotics, rapid advancements in language-based foundation models specifically are creating new possibilities for high-level decision modules during robotic control (Brown et al., 2020; Wang et al., 2024; Jeong et al., 2024). Using natural-language as an input coupled with advancements in language models, namely, large language models (LLMs), researchers have been able to leverage these inputs to describe a sequence of high-level actions for a robot to execute (Huang et al., 2022; Ahn et al., 2022; Song et al., 2023). Initial efforts have shown that off-the-shelf LLMs like GPT-3 can generate reasonable tasks in simulation for robotic control (Brown et al., 2020; Huang et al., 2022), with more recent work demonstrating full integration of LLMs with real robotics capable of using language models for reasoning and execution via low-level controllers (Ahn et al., 2022; Driess et al., 2023; Ding et al., 2023; Liang et al., 2023; Brohan et al., 2023). This speaks to a larger trend in combining foundation models with robotic planning, decision making, and execution (Wang et al., 2024; Jeong et al., 2024; Kawaharazuka et al., 2024). This sparks new questions around efficient design for incorporating language interfaces effectively.

It is critical to consider how to address well-established limitations in LLMs, such as their tendency for hallucinations (Chakraborty et al., 2025; Han et al., 2026), their limited capability in sequential decision making (Furuta et al., 2024), spatial understanding (Liu et al., 2025) and learning from experience and adapting to their environment (Zhou et al., 2023), when examining their role in robotic planning and execution. Limitations of LLMs can have compounding negative consequences when for robotics, where hallucinations could lead to unsafe or unpredictable behavior (Wang et al., 2024; Vemprala et al., 2024). Hybrid interface design which leverages the strengths of the language models with low-level controller information such as from sensors or action policies offer a potential solution to these concerns (Aghaee and Shaker, 2026; Darmanin and Vella, 2025).

A key design consideration in hybrid architectures is how low-level information is integrated and “shown” to the language model, as this can impact both the model’s policy and the controller’s actions (Wang et al., 2024; Yang et al., 2023). Rather than treating the language models as a black box, recent work has explored hybrid architectures utilizing reinforcement learning (RL) agents to work in tandem with the LLM in a variety of combined paradigms (Ahn et al., 2022; Liang et al., 2023; Carta et al., 2023; Hu et al., 2024). In the advisor–arbiter paradigm (Zhou et al., 2023; Asawa et al., 2025), a language model might suggest high-level actions or plans that a learned policy executes or vetoes, based on feasibility (Ahn et al., 2022; Hu et al., 2024). Conversely, a trained reinforcement policy can also serve as a “student” that learns from the actions of the language model, which can accelerate learning (Zhou et al., 2024). Others have proposed additional dual-policy or guidance frameworks where a guide policy from an LLM or a scripted planner provides the exploratory hints while a standard RL policy refines behavior in the system (Uchendu et al., 2023; Hu et al., 2024). Such approaches capitalize on a language model’s strategic reasoning strengths, while a RL agent handles low-level trial and error, potentially yielding more sample-efficient learning (Zhou et al., 2024; Du et al., 2023). Another variant of this dual-policy approach is using LLM-generated trajectories to pre-train an RL agent or to shape its reward function (Du et al., 2023; Colas et al., 2023; Yu et al., 2023). In these hybrid systems, prompt framing is often overlooked despite its potentially significant impact (Chen et al., 2025a).

While general prompt engineering has been well established to leverage LLMs sensitivity to phrasing and context, effective prompt framing can significantly influence an LLM’s performance on sequential decision tasks (Wei et al., 2022; Yao et al., 2022; Shinn et al., 2023). Prompt framing can affect how implicit specifications change a model’s interpretation of the same inputs and therefore how it chooses actions (Shanahan et al., 2023; Kim et al., 2024). For example, providing structured prompts (i.e., with added context or action templates) have enabled GPT-3 models to produce valid plans for embodied agents (Huang et al., 2022; Liu et al., 2024; Chen et al., 2025b). Additionally prompt framing and output formatting have been demonstrated to be helpful in robotic control, where structure can help ensure resulting plans align with the robot’s capabilities and task requirements (Liang et al., 2023; Vemprala et al., 2024). However, in many robotics and agentic workflows, prompt iteration is trial-and-error, and evaluation often focuses on single-turn correctness rather than closed-loop behavior (Vemprala et al., 2024; Xi et al., 2023). Sequential decision tasks provide objective metrics that directly reflect behavior under constraints, such as success rate, cumulative reward, and penalties from unsafe or inefficient actions (Dann and Brunskill, 2015; Silver et al., 2016; Yang et al., 2023). Critically, this viewpoint allows prompt changes to be evaluated as measurable interventions rather than stylistic preferences (Kong et al., 2024; Kim et al., 2024), where controlled prompt comparisons can be done. To effectively do these comparisons, it is critical to hold observation content, output constraints, and the evaluation protocol constant so that framing is the primary difference.

Here, we describe a platform for systematically investigating prompt framings and evaluating how they impact the performance of a combined LLM + RL agent, where the LLM’s involvement spans multiple interaction steps rather than a one-shot instruction. While a single prompt can yield a complete plan, it often cannot anticipate every contingency. This motivates iterative prompting frameworks in which the LLM and environment interact in a loop, and the model must consider not just the consequences of a single action but longer-horizon effects over multiple steps (Yao et al., 2022; Huang et al., 2023; Shinn et al., 2023). In these frameworks, the LLM can receive intermediate feedback (i.e., observations from the environment, signals on rewards, or success/failure outcomes) and revise its plan or suggest corrections. An example of this approach is the ReAct paradigm (described Yao et al., 2022), where the LLM interleaves reasoning statements and action commands step by step, and reflection approaches where the LLM pauses to reflect on errors after each attempt and then retries with an updated strategy. Brooks et al. similarly demonstrated that an LLM (Codex) can implement trial-and-error learning purely via in-context prompt updates, effectively performing policy iteration by updating its own prompt with new state–action examples after each episode. These findings reinforce that feedback loops and sequential prompts are important for decision-making when utilizing foundation models in robotics, in contrast to treating them as fixed one-shot, one-question agents (Xi et al., 2023; Yang et al., 2023). Therefore, in our described experimental platform, we first focus on how the LLM functions as an interactive decision module across multiple steps within a hybrid system.

**FIGURE 1:**
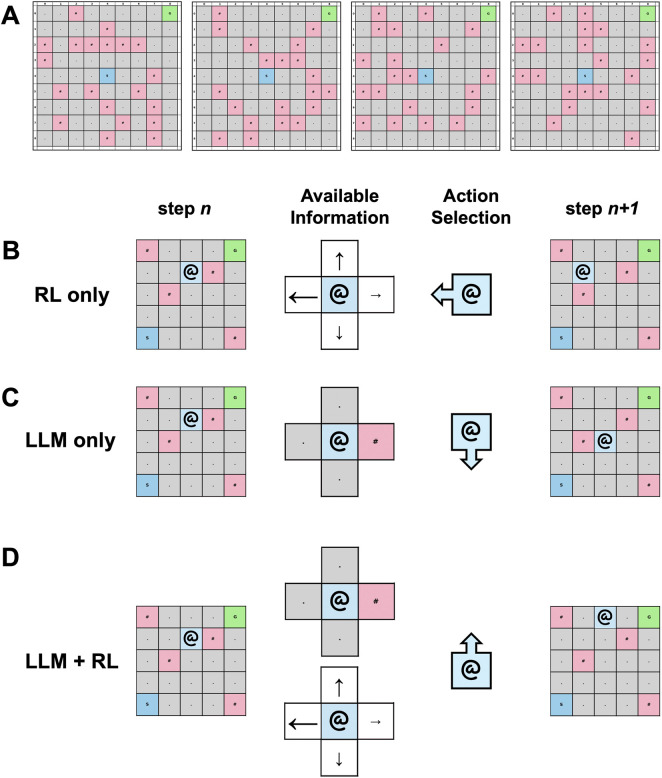
Gridworld environment for evaluating hybrid LLM + RL agents. **(A)** Examples of randomly generated Gridworld environments. **(B)** Decision process of RL-only agent for a given step. **(C)** Decision process of LLM-only agent for a given step. **(D)** Decision process of hybrid LLM + RL agent for a given step.

The primary contribution of this work is to present an initial investigation of how this controlled platform can be used to quantify how structured advice and prompt framing shape behavior in a hybrid LLM + RL control loop, using a Gridworld-based environment as a demonstrative and minimal navigation task (Figure 1A). The Gridworld is a well-established approach which provides a simplified proxy for indoor navigation with obstacles, local sensing, and repeated decision-making under a step budget (Kaelbling et al., 1998). At each step, the hybrid agent receives a local observation and an advisor recommendation, and the language model selects the executed action from a fixed set of four moves. While there is a significant gap between Gridworld and a full system for robotic navigation in indoor environments, this design choice was intentionally chosen to enable study of prompt- and interface-driven effects of a language foundation model using a simplified and representative advisor-arbiter hybrid control agent (Chevalier-Boisvert et al., 2019; Shridhar et al., 2021). The platform enables prompt-factor sweeps where only the role framing changes while observation content and action constraints remain unchanged. It allows enforcement of a strict output format so each step yields a valid action, and disagreement with the advisor can be measured reliably. Furthermore, it supports ablations that replace the advisor with random actions or with a null recommendation token, to test whether structured advice matters beyond the presence of extra text. These design choices aim to isolate conditional effects of prompting and advice channels, rather than to build a fully realistic navigation system.

**FIGURE 2:**
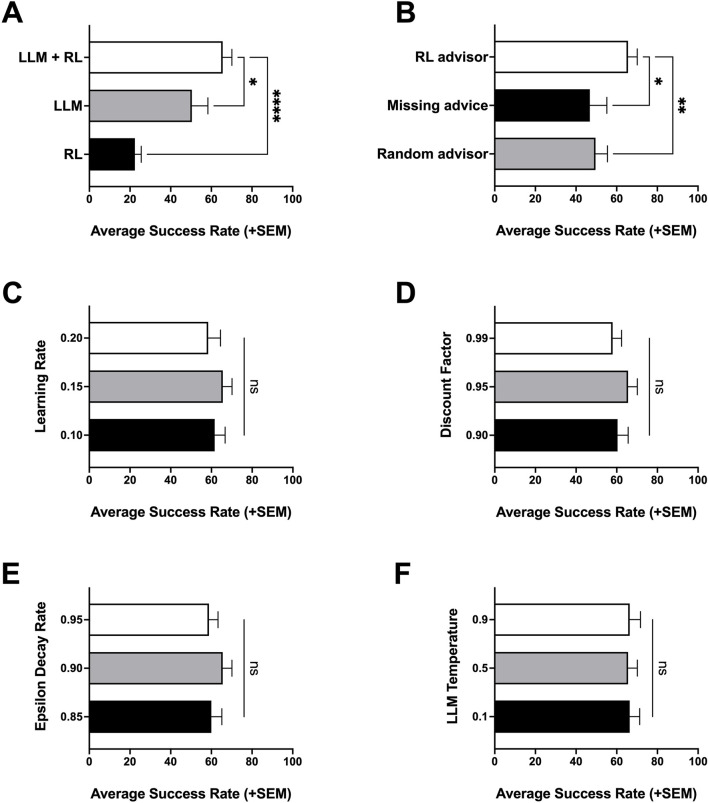
Baseline comparisons, ablations and sensitivity to RL hyperparameters of hybrid LLM + RL agent. **(A)** Success rate of RL-only, LLM-only and LLM + RL agent. **(B)** Changes in success rate of LLM + RL agent with ablations (missing advice, random advice). **(C)** Sensitivity of LLM + RL agent to changes in Q-learning RL policy’s learning rate hyperparameter. **(D)** Sensitivity of LLM + RL agent to changes in Q-learning RL policy’s discount factor hyperparameter. **(E)** Sensitivity of LLM + RL agent to changes in Q-learning RL policy’s epsilon decay rate hyperparameter. **(F)** Sensitivity of LLM + RL agent to changes in LLM model temperature inference parameter. Statistical significance was evaluated using a one-way repeated-measures ANOVA with Dunnett’s post hoc test for multiple-comparison and are indicated as: *p < 0.05; **p < 0.01; ****p < 0.0001; ns, not significant.

**FIGURE 3 F3:**
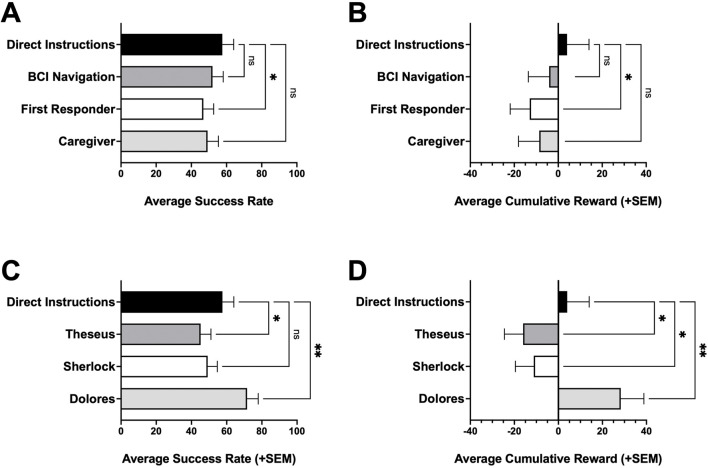
Evaluating performance of different LLM personas. **(A)** Average success rate of navigation persona agents. **(B)** Average cumulative reward of navigation persona agents. **(C)** Average success rate of narrative persona agents. **(D)** Average cumulative reward of narrative persona agents. Statistical significance was evaluated using a one-way repeated-measures ANOVA with Dunnett’s post hoc test for multiple-comparison and are indicated as: *p < 0.05; **p < 0.01; ns, not significant.

**FIGURE 4 F4:**
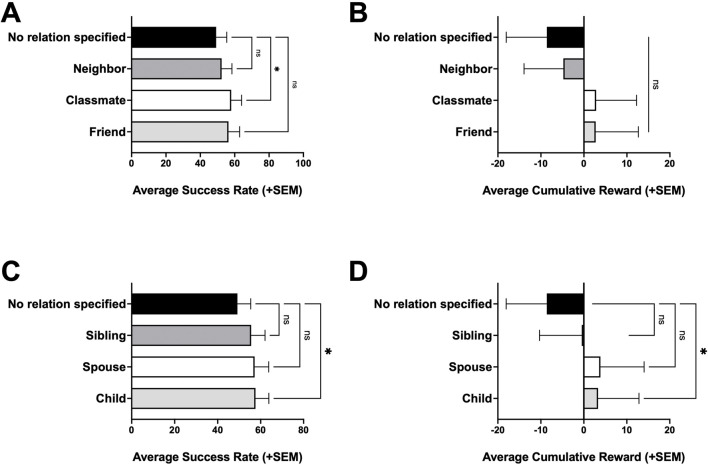
Investigating effect of relational framing on caregiver persona. **(A)** Average success rate with non-familial relational framing. **(B)** Average cumulative reward with non-familial relational framing. **(C)** Average success rate with familial relational framing. **(D)** Average cumulative reward with familial relational framing. Statistical significance was evaluated using a one-way repeated-measures ANOVA with Dunnett’s post hoc test for multiple-comparison and are indicated as: *p < 0.05; ns, not significant.

Using this platform, we compare performance of RL-only, LLM-only, and hybrid LLM + RL agents in a Gridworld environment (Figures 1B–D). We evaluate advisor-channel ablations and their effects and summarize sensitivity to key RL and LLM hyperparameters (Figure 2). We then demonstrate exploratory controlled experiments on the effects of prompt framing under different personas (Figure 3) and relational framing (Figure 4).

## 2 Methods

### 2.1 Overview of experimental platform

We developed a controlled experimental platform to quantify how LLM prompt framing (“persona” or role instructions) influences action selection when the LLM arbitrates an RL “advisor.” The platform has four core components: (i) a procedurally generated 2D Gridworld navigation environment, (ii) a tabular Q-learning agent, (iii) an LLM agent with a fixed input/output scaffold and strict parsing, and (iv) a hybrid LLM + RL agent that combines the RL policy and the LLM in an advisor–arbiter loop.

A central design goal is paired evaluation: world configurations and initial Q-tables are saved and reused so that all conditions can be compared under identical initial conditions (same obstacle layout, same start/goal, same initial Q-values). The platform includes step-level logging of state/action/reward transitions, the RL recommendation, the parsed LLM decision, and an “override” label indicating whether the LLM executed the RL recommendation or disagreed with it.

### 2.2 Gridworld task environment

The navigation environment is a square grid of configurable size constrained to odd number. The start position is fixed at the center cell, and the goal is fixed at the top-right corner cell. Obstacles are randomly placed at a specified density (nominally ∼25% of grid cells), and walls correspond to the grid boundary. Worlds are generated by randomly placing obstacles subject to validity constraints. Specifically, obstacle placement uses rejection sampling so that (i) there exists at least one path from start to goal and (ii) the free space is fully connected (no unreachable “dead zones”). Each accepted world is saved to disk (grid mask and start/goal positions), and the same saved world is reused across all agent conditions for paired evaluation. All primary experiments use a 9 × 9 grid. Additional experiments in Supplementary Figure S1 using 7 × 7, 11 × 11 and 13 × 13 grids generated in the same way.

The task objective is to reach the goal cell from the start cell while navigating around obstacles. The agent moves in a 4-connected action space (up/down/left/right); walls and obstacles prevent movement. An episode ends when (i) the goal is reached or (ii) a maximum step budget is reached. This maximum limit is set to the grid size (81) for all experiments. If an action would move the agent into a wall (out of bounds) or an obstacle, the agent remains in place and receives an invalid-move penalty. Rewards are assigned as: valid move step cost −1, wall/obstacle collision −10 (no movement), and reaching the goal +100 (terminal).

### 2.3 Agent architectures

We evaluate three agent classes: RL-only, LLM-only, and hybrid LLM + RL. All agents interact with the same environment and reward function; they differ only in what information they receive and how actions are chosen.

#### 2.3.1 RL-only

The RL-only baseline is a tabular Q-learning agent (Watkins and Dayan, 1992) whose Q-table is indexed by grid coordinate (row, col) and action (4 actions). During training, actions are selected using an ε-greedy policy with ε decaying across episodes. Q-values are initialized to small random values sampled independently from a uniform distribution between 0.0 and 0.1. To support paired comparisons, all RL-based agents (RL-only, LLM + RL and LLM + RL persona variants) in a given Gridworld start with the same randomly initialized Q-table. The RL hyperparameters used for tabular Q-learning were a learning rate of 0.15, discount factor of 0.95, initial epsilon of 0.95, minimum epsilon of 0.1 and epsilon decay rate of 0.9 for most experiments (Figures 2A,B,F,3,4; [Fig F3] [Fig F4] Supplementary Figures S1–S5). Experiments for Figures 2C–E tested the impact of additional RL hyperparameters, namely, learning rate (0.10 and 0.20), discount factor (0.90 and 0.99) and epsilon decay rate (0.85 and 0.95), respectively.

#### 2.3.2 LLM-only

The LLM-only baseline receives the same local observation ring (and position if enabled) and outputs one of four actions in a strictly constrained format. The LLM is stateless; it does not receive histories of past states or actions. The output is parsed from a fixed template; if parsing fails, a defined fallback behavior is used.

#### 2.3.3 LLM + RL

The hybrid agent is an advisor–arbiter architecture in which the RL module provides an action recommendation and the LLM selects the executed action. The per-step loop is: observe → RL recommends an action → LLM receives observation + recommendation → LLM outputs executed action → environment transitions → Q-table updates using the executed transition.

The advisor is a standard Q-learning policy queried at the current grid coordinate. During training, the advisor recommendation is ε-greedy: with probability ε it recommends a random action, otherwise it recommends the current best action under the Q-table. ε decays across episodes according to the reported schedule, shifting the advisor from mostly-random to mostly-greedy recommendations over the short training budget. The advisor’s Q-table is updated based on the executed action, not the advisor’s recommended action.

LLM arbiters share the same prompt scaffold: a system prompt that specifies a role framing (persona), and a user message that provides the observation fields and (for the hybrid) the advisor’s recommended action. The LLM produces (i) a short report and (ii) a final action in a fixed, machine-parsable format (e.g., XML-like tags), which is parsed into one of the four actions. The LLM configuration (model/provider and temperature) is held fixed across conditions and reported explicitly. Primary experiments (Figures 2–4) and Supplementary Figures 1, 2, 4, 5 were done using the GPT-OSS-120B on the Cerebras inference API. Supplementary Figure S3, which compares different LLM models used arbiters, used Llama-3.3-70B on the Cerebras inference API. The LLM inference temperature was set to 0.5 for all experiments (Figures 2A–E, 3, 4; Supplementary Figures S1–S5) expect for the experiment presented in Figure 2F, which tested additional LLM temperatures (0.1 and 0.9).

### 2.4 Experiments

Experiments are designed to (i) establish baseline performance differences among RL-only, LLM-only, and LLM + RL, (ii) test whether structured RL advice matters via ablations, (iii) characterize sensitivity to key RL hyperparameters, and (iv) quantify LLM persona-driven differences under matched conditions.

Experiments are repeated across multiple randomly initialized Gridworlds. Within an experimental run, all agent conditions and personas start from the same world and the same initial Q-table, where relevant, to enable paired comparisons across prompts and agent architectures. Each agent is executed under a short training budget (10 episodes per world), and performance during training is measured over those same episodes. Additional experiments in Supplementary Figure S2 used an extended training budget (25 episodes per world).

Across the study, we generated 50 total worlds: 25 for the baseline, ablation, and sensitivity experiments (Figure 2; Supplementary Figures S1–S3) and an additional 25 for persona conditioning and caregiver relationship variants (Figures 3, 4; Supplementary Figures S4–S5).

### 2.5 LLM personas

We investigated multiple personas, or sets of system and user instructions given to the LLM arbiter: observation formatting, decision constraints, and parsing remain constant across personas. Personas are grouped into navigation-relevant roles and narrative/story roles to align with assistive-navigation and decision-making contexts in robotics.

#### 2.5.1 Navigation personas

Navigation personas are intended to resemble applied robotics contexts, such as a BCI navigation module, a first responder operating a remote vehicle, or an attentive caregiver assisting a wheelchair user. These are interpreted relative to the “Direct Instructions” baseline to evaluate whether role framing changes decision-making under identical sensory inputs and advisor signals.

#### 2.5.2 Narrative personas

Narrative personas are fictional framings drawn from literary sources (e.g., Theseus in a labyrinth, Sherlock in a maze, Westworld-inspired “Dolores”), with narrative-relevant task framing and instruction prompting changes. These framings are evaluated under the same observation and action constraints to avoid confounding narrative with additional information.

#### 2.5.3 Caregiver persona relationships

To probe whether relational framing induces systematic changes within a single navigation role, we created caregiver variants that modify only the relationship label (e.g., “you are helping an individual” becomes “you are helping your neighbor/friend/child/spouse” while keeping all other task instructions fixed.

### 2.6 Metrics and statistical analysis

The primary metrics evaluated are average success rate in the 10-episode training budget and average cumulative reward at the end of a training episode. A secondary metric, the average number of steps taken in successful trials, is presented in Supplementary Figures S4–S5. All analyses use the paired-by-world experimental design: each world contributes matched measurements across conditions, enabling repeated-measures inference. We use one-way repeated-measures ANOVA with Dunnett’s *post hoc* test for multiple-comparisons.

## 3 Results

### 3.1 Hybrid LLM + RL agent improves gridworld success rate across short training windows

Figure 1 summarizes the grid-world navigation environment and the core agent variants evaluated in this study. Figure 1A shows examples of randomly initialized grid worlds in this navigation task. Episodes end when the agent either reaches the goal or exhausts the episode’s step budget. Figures 1B–D summarizes the three agent configurations evaluated. The RL-only agent is a tabular Q-learning agent that uses the agent’s current grid cell (row, col) as its state and selects an action from its Q-table, executes that action, and transitions to the next state (Figure 1B).

An LLM-only agent is told it is performing a grid navigation task and only receives a description of its sensor readings in its immediate vicinity–in other words, local observations describing the neighboring cell types in the four cardinal directions relative to the current position (Figure 1C). These local observations tell the agent whether the neighboring cell is empty, a wall, an obstacle, the starting position or the goal. The LLM-only agent makes an action selection based on its task instructions and local observations, without any RL policy recommendations.

The LLM + RL hybrid agent works as follows: the RL policy provides an action suggestion and the LLM is the final arbiter of the executed action (Figure 1D). As in the LLM-only agent, the LLM in the hybrid agent is told it is performing a grid navigation task and receives local observations. Additionally, it receives the RL policy’s action suggestion framed as recommendations from an experience-based learning system. In this study the RL policy is a tabular Q-learning agent, as in the RL-only agent. The Q-learning updates of the RL policy in the hybrid agent are applied using the actual state-action transitions executed by the LLM, not the RL policy’s suggested actions. Across the 10-episode training window evaluated in this study, the hybrid LLM + RL agent achieved significantly higher success rate compared to the RL-only agent (p < 0.001) and LLM -only agent (p < 0.05) (Figure 2A). The same effect was observed when this was repeated on smaller (7 × 7) and larger grids (11 × 11, 13 × 13) (Supplementary Figure S1), and across longer training budgets (25 episodes) (Supplementary Figure S2).

### 3.2 Ablation testing of hybrid LLM + RL agents

We evaluated ablations to the RL advisor policy to test whether improvements in the hybrid LLM + RL agent are attributable to structured RL-derived suggestions rather than simply the presence of additional input to the LLM (Figure 2B).

The first ablation to the hybrid agent was a random advisor, where the LLM still receives action suggestions but, rather than being pulled from an RL policy, they are randomly selected from the four available actions. This condition preserves the presence of an advisory channel but removes the learned recommendations from the RL policy. The second ablation is one where the LLM is expecting an input, but the recommendation field is set to “None” instead of providing a suggested action. This evaluates what the model does in the absence of a suggestion when it expects one. Both ablations perform worse than the original hybrid LLM + RL agent with the Q-learning policy, indicating that the performance of the hybrid LLM + RL agent are not attributable solely to the presence of additional text or to random suggestions.

### 3.3 Hybrid LLM + RL agent sensitivity to hyperparameters

To assess robustness of the hybrid LLM + RL agent to RL and LLM hyperparameters we performed a brief sensitivity analysis. The key RL training hyperparameters evaluated for the Q-learning agent used were the learning rate, discount factor, and the epsilon-greedy decay rate (Figures 2C–E). For LLM inference hyperparameters, we evaluated different configurations for LLM inference, namely, the temperature parameter (Figure 2F) and the model family (Supplementary Figure S3). Overall, these hyperparameters introduce a modest effect on how well the LLM + RL agent is able to utilize the advisor, reflected in small changes in success rate.

### 3.4 LLM persona conditioning

We explored how persona conditioning (i.e., alternate contextual framing) changes performance of this hybrid LLM + RL agent (Figure 3). The underlying hybrid architecture is held constant: the LLM receives recommendations from a Q-learning policy. What changes is contextual task framing role-based prompting. The persona framing of the task is varied while keeping the underlying agent architecture—including the RL policy, training environment, and training budget—constant. These experiments were done on a new set of 25 initialized worlds. Within this persona experiment set, comparisons are paired by world so that each persona is evaluated on the same worlds under the same initialization protocol, including the same initial Q-table. The persona framings fall into two broad categories: navigation-context personas intended to resemble applied robotics contexts (e.g., a control module for a BCI-controlled power wheelchair, a first responder operating a remote robotic vehicle, or a caregiver physically pushing someone in a wheelchair), and narrative personas intended to provide a story-based framing while keeping the task identical. The narrative framings evaluated include Theseus in a labyrinth, Sherlock in a maze, and a Westworld-inspired “Dolores” agent in a labyrinth lab experiment.

Navigation-context personas showed similar or slightly worse performance than the baseline model, which received direct task instructions (Figures 3A,B). In contrast, narrative personas showed more variability in task performance (Figures 3C,D). In particular, the Theseus persona showed a statistically significant decrease in success rate and mean episode reward (p < 0.05), and the Dolores agent showed marked improvement in success rate and mean reward relative to baseline hybrid instructions (p < 0.01). This improvement in the Dolores narrative persona agent was reflected in the average path length taken by the agent (Supplementary Figure S4).

### 3.5 Relational framing within the caregiver persona

Finally, we explored how interpersonal relational framing of the task may impact performance of persona-conditioned hybrid agents (Figure 4). For these experiments we focus on the caregiver persona, which has no relation specified in the baseline persona; the model is instructed that it is helping an individual in a wheelchair navigate toward their destination in an indoor environment. To modify the relational framing, we simply change the instruction to reframe the individual as someone the caregiver has as a social relation with. In other words, the only change in the instructions is the relationship between the caregiver and the person being helped, e.g., instead of “a caregiver assisting an individual in a wheelchair” in the baseline caregiver persona, the LLM is instructed that it is assisting a neighbor, classmate, friend, cousin, sibling, child, or spouse in a wheelchair in these caregiver persona variants.

In the limited number of relational framing caregiver persona variants we evaluated, we see that these variants had modest increases in average success rate and average cumulative reward. Overall, the more familiar relationships show higher success, with a statistically significant improvement of average success rate and cumulative reward in the “caregiver helping their child” variant (p < 0.05). However, this trend toward improved performance with more familiar relationships was not reflected in the average path length taken by the caregiver persona variants (Supplementary Figure S5).

## 4 Discussion

This work demonstrates key initial experiments exploring a platform for controlled and structured evaluation of dual-policy LLM + RL agents. The initial findings presented show the platform is effective in assessing differences in prompt framing and ablation across several scenarios using a Gridworld navigation task as a minimal simulation of indoor robotic navigation. Our initial experiments validated this platform using a short interaction budget of 10 episodes per world to emphasize early behavior under constrained resources rather than asymptotic reinforcement learning performance (Dann and Brunskill, 2015; Saunders et al., 2018). This choice was motivated pragmatically by foundation-model runtime and cost, and it also reflects settings where interaction and resets are limited by safety, time, or operational constraints such as high computational demand (Saunders et al., 2018; Wang et al., 2024) and energy costs of LLMs (Strubell et al., 2019; Luccioni et al., 2024; Fernandez et al., 2025). Additionally, the LLM was treated as stateless to simplify interpretation and isolate prompt-conditioned action selection, not to model realistic memory-based navigation (Brooks et al., 2023).

The findings shown in our first experiment (Figure 2A) demonstrate that within the controlled Gridworld testbed, the hybrid LLM + RL advisor–arbiter agent achieved higher short-budget performance than either an RL-only agent or an LLM-only agent. This result is in line with expectations from other reports in the literature where hybrid agents can utilize external knowledge (i.e., foundation models such as LLMs) can improve an RL agent’s early performance (Ahn et al., 2022; Wu et al., 2025). This finding helps reinforce that the LLM arbiter can inject high-level priors or natural language intuitions that can help overcome myopic or random initial behavior typical in RL agents, while still enabling the agent to learn from other environmental feedback.

Outcomes from the ablation experiments indicate that the hybrid LLM + RL agent’s advantage was not merely due to the presence of additional input channel of information (Figure 2B). Instead, the findings indicate that the content of the RL policy suggestion played an active role in contributing to the overall improvement of the hybrid agent as compared to the LLM-only and RL-only agents. This observation is again in line with prior studies on action advising and policy shaping, where random action suggestions or no advisor input can lead to little or no gain in learning efficiency (Torrey and Taylor, 2013; Griffith et al., 2013). In our case, the RL policy provided nontrivial structured recommendations that the LLM could exploit in an iterative looping fashion. The LLM arbiter was able to interpret when to follow or override these suggestions, presumably using its world knowledge and the immediate sensory context. The poor outcomes of the “random advisor” and “no advisor” conditions reinforce that the performance gains are not attributable to extra prompt tokens or some generic regularization effect, but to the substance of the RL-driven advice. This finding resonates with the concept of policy shaping by a knowledgeable teacher where the advisor’s input biased the arbiter toward better actions than it would have taken on its own, much as informative human feedback can bias an RL policy toward optimal decisions (Griffith et al., 2013).

The persona and relational experiments serve primarily as demonstrative examples of how this platform can vary prompt framing in a controlled way and measure which variations matter (Figures 3, 4). Persona and role instructions are a natural first dimension to study on this platform, because they are commonly used and can be varied without changing the task dynamics (Shanahan et al., 2023; Kong et al., 2024; Deshpande et al., 2023). In our experiments, the navigation-role prompts were designed to resemble applied contexts such as assistive navigation modules, first-response teleoperation, and caregiving, while keeping the observation and action constraints identical. Narrative prompts added story context while preserving the same underlying task. Importantly, these different personas received the same observations and advisor suggestions in our experimental platform, making the initial role description in the LLM’s prompt as the only critical piece of information to change. Despite this minimal difference, the agent’s decision patterns measurably shifted with different persona prompting. The observed heterogeneity from these personas suggests that some framings may introduce priors that align with the task and improve consistency, whereas other framings may introduce caution, competing goals, or interpretation biases that degrade performance. Relational wording within the caregiver prompt similarly illustrates that small, semantically meaningful modifications can correlate with measurable performance differences when using LLMs in practice. We emphasize that these prompt-framing results are intended as controlled measurements and examples of how this platform can be used as a testbed to investigate how structured advice and prompt framing shape behavior in a hybrid LLM + RL control loop, not as causal explanations of why a given role helps or hurts. For example, the path length summaries (average steps on successful episodes, Supplementary Figures S4, S5) illustrate one simple diagnostic the platform supports for follow-up: they help distinguish prompt effects that primarily improve efficiency (shorter successful trajectories) from effects that primarily reduce failure modes (higher success without shorter paths). The platform’s purpose is to enable more targeted follow-up analyses (e.g., rates of invalid moves, revisits/loops, or other error modes) in future work, rather than to claim a mechanistic account from these initial experiments.

Taken together, the results in this work show that prompt framing can measurably change a foundation model’s action selection in a fixed control loop. Our findings support the theory that the policy guiding LLM-driven action selection is not fixed, and consequently, can be steered by factors such as persona-driven task framing or relational context. They align well with observations from prior work that LLM-based agents can be guided via natural language instructions or “personas” to adopt certain strategies (Reed, 2022; Park et al., 2023), even if those strategies are not explicitly learned from the environment. In conjunction with previous exploration int this topic, narrative and relational framing may be useful for both task and performance optimization as well as for value alignment in multi-step decision making processes. Importantly, however, our findings also emphasize a need for developing guidelines for reliable persona design when deploying LLM-based decision arbiters, as the influence of fictional or non-task-grounded prompt frames could lead to unpredictability and (in some cases) personas making suboptimal choices.

As the purpose of this study is to present a novel experimental platform that enables a structured approach to investigating the effect of prompt framing, several study design choices were intentionally minimal. The environment is a discrete Gridworld with simplified dynamics and a local observation ring, and it does not capture continuous control, rich sensing, or uncertainty in state estimation. The advisor is tabular Q-learning, which does not address generalization through function approximation. The language model is stateless across steps, which isolates conditional prompt effects but does not test memory, long-horizon planning, or belief-state tracking. In stateful or history-augmented hybrid LLM + RL agents, framing effects may persist or evolve across steps because the model’s context includes prior observations and decisions, potentially changing both the magnitude and stability of persona-driven differences compared to the stateless setting. Results may also depend on the chosen language model and decoding settings, making cross-model robustness an open question. Additionally, the prompt comparisons in relational variants may be underpowered for small effect sizes. Finally, the metrics emphasize task completion and efficiency proxies rather than robotics-specific criteria such as comfort, smoothness, or safety beyond obstacle avoidance.

Despite its simplicity, the platform outlined in this work represents an important tool for practical design in robotics looking to leverage language-based foundation models. Our platform demonstrates how prompts and model interfaces can be treated as measurable system parameters and tested under controlled, paired conditions. Our work demonstrates the importance of evaluating these prompt framings, as they can have measurable impacts on outcomes. A direct next step is to add memory or stateful controllers while keeping the same interface and logging, to test whether prompt effects persist when history is available. Another line of research of interest is to replace tabular RL with function approximation and richer observations, while preserving the advisor–arbiter architecture. The advisor channel can be generalized beyond RL to planners, model-predictive control, safety modules, or human guidance, allowing studies of shared autonomy using the same measurement approach. Moving the task into richer navigation simulators would also increase realism while retaining paired-world evaluation and arbitration diagnostics. Finally, cross-model benchmarking would help quantify how prompt sensitivity depends on model family and decoding settings.

## Data Availability

The raw data supporting the conclusions of this article will be made available by the authors, without undue reservation.
