# Supplementary material for: A platform for investigating prompt framing as interface parameters in foundation models for robotics
Source: Front Robot AI. 2026 Apr 22;13:1771992. doi: 10.3389/frobt.2026.1771992 (PMC13143583; doi:10.3389/frobt.2026.1771992)
Supplement: Supplementary file 1 [file DataSheet1.docx]

Supplementary Material

**Supplemental Figure 1.** Success rate of RL-only, LLM-only and LLM+RL agents on
(A) 7×7 (N=25), (B) 11×11 (N=25) and (C) 13×113 (N=25) Gridworlds. All other experimental settings were the same as in main experiments (LLM model and temperature, RL hyperparameters, number of episodes). Statistical analysis done using a paired-by-world design and one-way repeated-measures ANOVA with Dunnett’s post‑hoc test for multiple comparisons.

**Supplemental Figure 2.** Success rate of RL-only, LLM-only and LLM+RL agents over a longer episode horizon (25 episodes) on 9×9 Gridworlds (N=25). All other experimental settings were the same as in main experiments (LLM model and temperature, RL hyperparameters). Statistical analysis done using a paired-by-world design and one-way repeated-measures ANOVA with Dunnett’s post‑hoc test for multiple comparisons.

**Supplemental Figure 3.** Success rate of LLM+RL agents when using an alternate LLM model (Llama-3.3 70B) from main experiments (GPT-OSS 120B) on 9 x 9 Gridworlds (N=25). All other experimental settings were the same as in main experiments (LLM temperature, RL hyperparameters, number of episodes).

**Supplemental Figure 4.** Average number of steps taken in successful trials by (A) Navigation persona agents (N=21-24)and (B) Narrative persona agents (N=22-24).

**Supplemental Figure 5.** Average number of steps taken in successful trial in Caregiver persona variants with (A) Non-familial relational framing (N=22-24) and (B) Familial relational framing (N=22-25).
